# Effects of Fillet Weld Size and Sleeve Material Strength on the Residual Stress Distribution and Structural Safety While Implementing the New Sleeve Repair Process

**DOI:** 10.3390/ma14237463

**Published:** 2021-12-05

**Authors:** Hongjie Zhang, Tao Han, Yong Wang, Qian Wu

**Affiliations:** 1 School of Materials Science and Engineering, China University of Petroleum, West Changjiang Road, Huangdao District, Qingdao 266580, China; thezhanghongjie@163.com (H.Z.); wangyong@upc.edu.cn (Y.W.); 2 Risk Assessment Institute, SINOPEC Research Institute of Safety Engineering, Co., Ltd., Songling Road, Laoshan District, Qingdao 266580, China; wuq.qday@sinopec.com

**Keywords:** in-service repair welding, fillet weld size, sleeve material strength, welding residual stress

## Abstract

The process optimization and structural safety improvement of the in-service repair welding of the X80 pipeline are very important. In this paper, the temperature, microstructure, and stress distribution were analyzed using the combination of TMM (thermal-metallurgical-mechanical) simulations and the corresponding verification experiments. The effects of the sleeve material strength and the fillet weld size were discussed. The results showed that the fillet weld zone was mainly composed of ferrite and bainite when the material of the sleeve pipe was Q345B. Furthermore, the sleeve pipe’s HAZ (heat affected zone) was dominated by lath martensite, lath bainite, and granular bainite. Moreover, granular bainite and a small amount of ferrite were found in the HAZ of the X80 pipe. It was found that, as the fillet weld size increased, the welding residual stress distribution became more uniform. The hoop stress at weld toe reduced from ~860 MPa of case A to ~680 MPa of case E, and the axial stress at weld toe reduced from ~440 MPa of case A to ~380 MPa of case E. From the viewpoint of welding residual stress, fillet weld size was suggested to be larger than 1.4T. The stress concentration and the stress distribution showed a correlation with the cracking behavior. Weld re-solidification ripples on the weld surface and weld ripples between welding passes or near the weld toe could cause stress concentration and the corresponding crack initiation. Furthermore, when the material of the sleeve pipe changed from Q345B to X80, the high-level tensile stress zone was found to be enlarged. The hoop stress at weld toe increased from ~750 to ~800 MPa, and the axial stress at weld toe increased from ~500 to ~600 MPa. After implementing the new sleeve repair welding process where X80 replaces the material of sleeve pipe, the cracking risk in sleeve pipe will improve. From the perspective of the welding residual stress, it was concluded that the fillet weld size reduction and the sleeve material strength improvement are harmful to in-service welded structures’ safety and integrity.

## 1. Introduction

In order to meet the energy demands and reduce the natural gas pipeline construction costs, X80 steel is widely used in China [1]. In contrast, the high-strength steel, exceeding X70, is rarely used in other countries. The investigations on the weldability and the crack-resistant ability [2,3,4,5] of X80 require further exploration. When compared to the initial welds, the repair welds were usually subjected to the multiaxial stress state, severe restraints in both hoop and axial directions, worse welding preparation, and welding environment [6,7]. Hence, the in-service repair welding of the X80 pipeline presents more challenges than the welding during the layout stage.

As far as in-service welding is concerned, two fundamental problems have to be investigated. One is the burn-through risk caused by the high temperature and corresponding material softening induced by welding. The other is the hydrogen-induced cracking after in-service welding. As reported in API 1104 [8], burn-through is not likely to happen when the wall thickness is greater than 6.4 mm. Generally, the wall thicknesses of the X80 pipeline are quite a bit larger than 6.4 mm. However, hydrogen-induced cracking becomes more accessible as the steel grade level increases. Furthermore, it is well known that there are three favorable factors to hydrogen-induced cracking namely hydrogen, susceptible microstructure, and residual stress [9]. The low-hydrogen electrode was demonstrated to effectively prevent hydrogen from being introduced to the welding joint [10]. By adopting the low-hydrogen electrode, the effects of hydrogen could be ignored. The microstructure is dependent on the welding thermal cycles. Hence, optimizing the susceptible microstructure is challenging for in-service repair welding. During the in-service welding, flowing natural gas in the pipeline dissipates a lot of welding heat and generates a high cooling rate [11]. Considering the preheating, and appropriately improving the heat input, usually show an insignificant effect on the welding thermal cycles. In the literature, several investigations on the susceptible microstructure were carried out [1].

However, there are only some investigations carried out on the in-service welding residual stress of X80. The impacts of residual stress on the hydrogen embrittlement are often ignored. The residual stress distributions of the repaired weld joints are uncomprehending for the constructors. However, many failure cases were related to the high-level residual stresses [12,13,14]. The in-service welding is usually subjected to the multiaxial stress state, i.e., pipe pressure and additional stress, and severe restraints in both hoop and axial direction [6,7]. Moreover, accurate residual stress prediction has become challenging.

According to API 1104 [8] and GB/T 31032 [15], Q345B/R/C should be considered to be the material of the sleeve pipe in the case of the sleeve repair welding of the X80 pipeline. The wall thickness of the sleeve pipe was usually thicker than that of the X80 pipeline, and the value might reach 60 mm. For security reasons, the fillet weld sizes were traditionally designed as large as possible. According to ASME [16], the fillet weld size should be 1.4 times the thickness of the pipeline that was to be repaired. Moreover, the other parts in the direction of the thickness of the sleeve pipe should be chamfered at a 45° angle. Furthermore, GB/T 28055 [17] and SYT6150 [18] indicated that the fillet weld size should be 2.0 times the wall thickness of the pipeline that was to be repaired. The latter was widely adopted for the in-service repair welding of the pipeline, as to the X80 pipeline, which referred to an amount of the welding workload. Moreover, the quality of the welding decreases as the number of welding passes increased. In order to address the above problem, replacing the sleeve material with higher-level steel, i.e., X80 steel, and reducing the fillet weld size obtained more attention. Presently, this new process is still at the developing stage. In this work, investigations were carried out to provide the temperature-microstructure-residual stress distributions in the repaired X80 weld joint, and to provide guidance for implementing the new process. Furthermore, the effects of fillet weld size on the residual stress distribution and the structure safety were also analyzed. Moreover, the effects of replacing the sleeve material from Q345B/R/C to X80 were discussed.

## 2. Materials and Experimental Procedure

### 2.1. Material and Experimental Process Description

**Figure 1:**
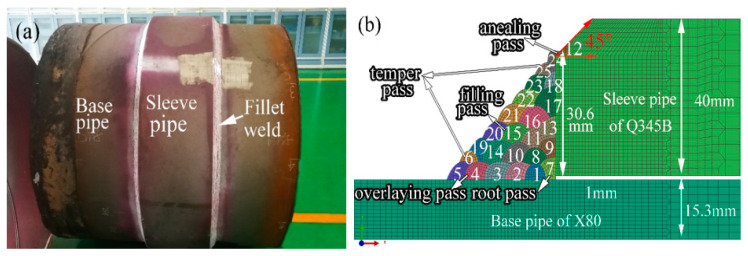
Schematic representation of type-B sleeve repair welding: (**a**) mock-up, (**b**) fillet weld.

**Figure 2:**
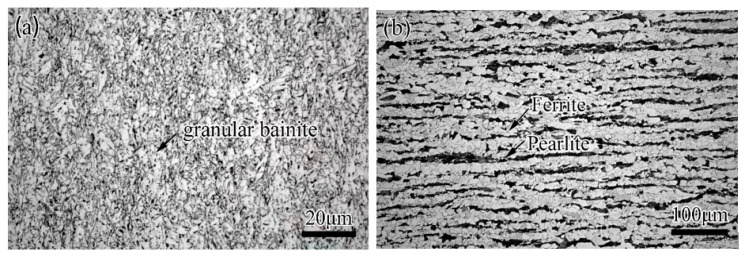
Microstructure of the base and sleeve pipe: (**a**) X80, (**b**) Q345B.

As given in Figure 1, the X80 pipe with an external diameter of 1219 mm and a wall thickness of 15.3 mm was the pipe that remained to repair. The microstructure of X80 mainly consisted of granular bainite (Figure 2a). The material of the sleeve pipe was Q345B, which consisted of ferrite and pearlite (Figure 2b). The external diameter and the wall thickness of the sleeve pipe were 1259 mm and 40 mm, respectively. The fillet weld size was twice the wall thickness of the base pipe (X80 pipe), and other parts in the thickness direction of the sleeve pipe were chamfered at a 45° angle (Figure 1b). The fillet girth welds consisted of 25 passes, and the deposition sequences were given in Figure 1b. The welding process was shielded metal arc welding (SMAW). A low-hydrogen electrode of E5515-G with a diameter of 3.2 mm was used in this investigation. The chemical compositions of X80, Q345B, and the E5515-G were listed in Table 1.

The mechanical properties of X80 and deposited metal of E5515-G were listed in Table 2.

The low-hydrogen electrodes were dried before welding as per the dry-out procedure, and the pressure of the pipe was maintained at 7 MPa during in-service welding. According to the procedure requirements, preheating and inter-pass temperatures should be over 60 °C and 80 °C, respectively. The overlaying passes were directly welded onto the X80 pipe in small heat input in order to avoid the burn-though, as shown in Figure 1. The seventh welding pass was found to be the root pass and used to connect the sleeve pipe and the X80 pipe. The filling, temper, and annealing passes are also shown in Figure 1. The detailed welding parameters are given in Table 3.

### 2.2. Verification Experiments

In this work, the temperature, microstructure, and stress field distributions were obtained using the SYSWELD simulation (SYSWELD 2008, ESI GROUP, PAIRS, FRANCE), and the results were verified using the experimental methods. The weld joint samples were etched with 4% Nital solution. The microstructures were characterized by the optical microscope (Leica DM2500 M, Leica Microsystems, ShangHai, China). The hardness of the welding joint was measured using a Vickers hardness tester (HVS-50, Beijing ShiDaiZhiFeng Instrument Co., Ltd., Beijing, China), with a force of 10 kgf (HV10) and a dwell time of 10 s. The welding residual stress was measured by the hole-drilling strain gauge method (HDM) [19].

## 3. Finite Element Modeling

**Figure 3:**
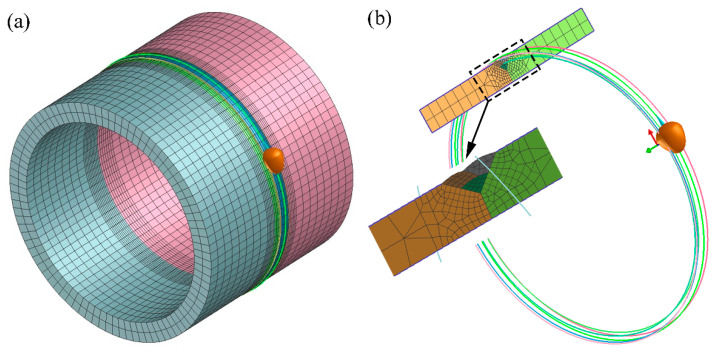
Schematic diagram of verification model: (**a**) 3D model, (**b**) 2D rotational model.

A thermal-metallurgical-mechanical (TMM) model was established to predict the temperature, microstructure, and stress field distributions. In this research, a 2D rotational model was chosen to balance the simulation accuracy and efficiency. The 2D rotational model considered all the boundary conditions for rotational structures like the pipe and obtained the same results as a 3D pipe structure. The 2D rotational model was widely adopted to replace the 3D model during the large-scale structure simulation. Compared to the 3D model, the reliability of the 2D rotational or 2D axisymmetric model was fully validated [20,21,22,23,24]. In order to demonstrate that the 2D rotational simulation process was reliable, a small-scale model was carried out to compare the 2D rotational simulation results with 3D results. The schematic diagram of the verification model was given, as shown in Figure 3.

**Figure 4:**
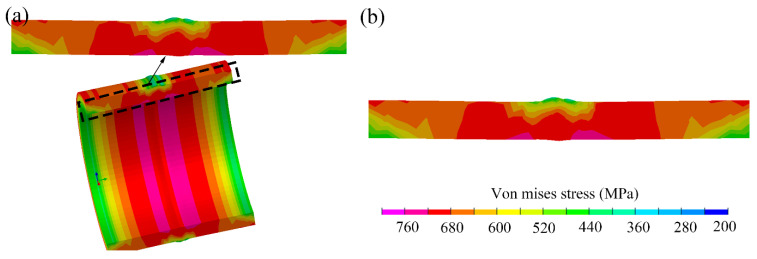
The von Mises stress contour: (**a**) 2D rotational simulation result, (**b**) 3D simulation result.

The results of 2D and 3D simulations were brought under the same welding conditions. As shown in Figure 4, the von Mises stress contour of the 2D rotational simulation was compared with that of 3D simulation. It can be found that the 2D results show good accuracy compared with the 3D results.

The in-service sleeve repair welding model (Figure 1) was built according to the actual weld joint. By considering the different requirements in the standards [16,17,18], the fillet weld size was found to affect structural safety significantly. The implementation of the new procure in the X80 pipeline by replacing the sleeve material with the higher level grade steel (X80) and reducing the welding passes is imperative in the future. Hence, the new procedure was designed to clarify the effects of the fillet weld size on the temperature-microstructure-stress fields and the structural safety.

### 3.1. Finite Element Model

**Figure 5:**
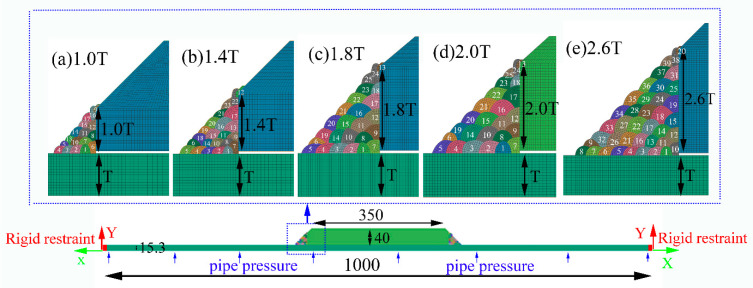
Finite element model of sleeve repair welding (mm): (**a**) 1.0T, (**b**) 1.4 T, (**c**) 1.8 T, (**d**) 2.0 T, (**e**) 2.6 T.

The fillet weld sizes, ranging from 1.0 times to 2.6 times the wall thickness of X80 pipe, were considered in this investigation. Accordingly, the finite element models were built, as shown in Figure 5. The sequence and the number of the welding passes are marked in the picture. The 45° chamfers were all implemented in these cases. Finite element models have meshed with hexahedron cubic elements. To balance the accuracy and efficiency, coarser meshes were used in the regions away from the weld zone, and finer meshes were used near the weld zone, with the smallest element size of 0.5 mm × 0.7mm. The total element number was 10,566. Rigid restraints were applied at the ends of the X80 pipe, as shown in Figure 5. The pipe pressure, which was perpendicular to the pipe’s inner surface, was implemented onto the inner surface based on the local coordinate system, as illustrated in Figure 5.

### 3.2. Thermal Analysis

The double ellipsoid heat source [25] was demonstrated to be appropriate to describe the heat flux distribution of SMAW, and the equations of the double ellipsoid heat source were given below:

(1) $$q_{f}(x,y,z)=\frac{12\sqrt{3}\eta UI}{(a_{f}+a_{r})b_{h}c_{h}\pi \sqrt{\pi }}\exp(-\frac{3x^{2}}{a_{f}^{2}}-\frac{3y^{2}}{b_{n}^{2}}-\frac{3z^{2}}{c_{h}^{2}}),x\geq 0$$

(2) $$q_{f}(x,y,z)=\frac{12\sqrt{3}\eta UI}{(a_{f}+a_{r})b_{h}c_{h}\pi \sqrt{\pi }}\exp(-\frac{3x^{2}}{a_{r}^{2}}-\frac{3y^{2}}{b_{n}^{2}}-\frac{3z^{2}}{c_{h}^{2}}),x\leq 0$$

where $q_{f}$ and $q_{r}$ are the power density functions (W m^−3^), η is the arc efficient, U is the arc voltage (V), I is the welding current (A), $a_{f}$, $a_{r}$, $\;b_{h}$ and $c_{h}$ are the distribution parameters.

**Figure 6:**
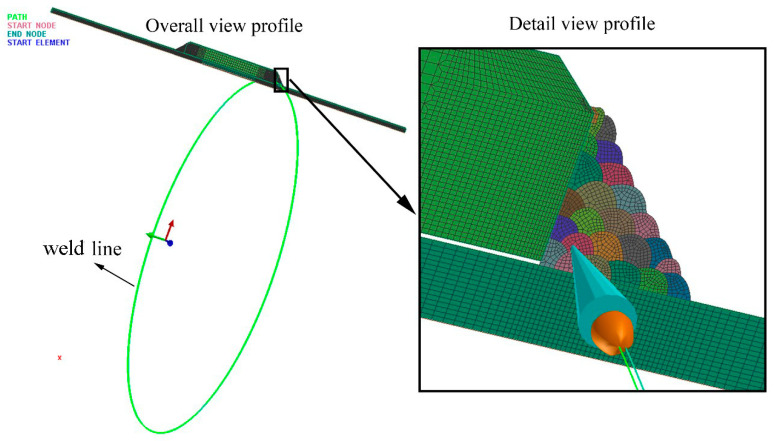
Schematic diagram of the 2D rotational model.

During the 2D rotational finite element modeling, the weld line was created following the actual welding trajectory. This weld line would tell the double ellipsoid heat source running along the weld line, as shown in Figure 6.

During in-service sleeve repair welding, the heat dissipation between the outer surface and air was usually treated as the natural convective heat transfer. In contrast, heat dissipation between the X80 pipe’s inner surface and the flowing natural gas was defined as the forced convection heat dissipation. Natural convective heat transfer mainly consists of radiation and natural convection. The governing equation is expressed below:

(3) $$h_{outer}=-5.67\times 10^{-8}\times (T+T_{0})(T^{2}+T_{0}^{2})+h_{c}$$

where $T_{0}$ is the room temperature (K), $h_{c}$ (25 W m^−2^ K^−1^) is the convection coefficient.

The governing equation of forced convection heat dissipation was reported in our previous studies [26] and given below:

(4) hinner=0.8×5.67×10−8[(273.15+T0)+(273.15+T)]⋅[(273.15+T0)2+(273.15+T)2]+0.027ρfλfRef0.8Prf1/31.8165μf0.14μ00.14(273.15+T)0.1064

where $\rho_{f}$ is the gas density, $\lambda_{f}$ is the heat conductivity coefficient, $\mu_{0}$ and $\mu_{f}$ are the kinematic viscosity coefficients, ${\mathrm{Pr}}_{f}$ is the Prandtl number, Ref is the Reynolds number.

Non-linear transient heat transfer equation was expressed as follows [27]:

(5) $$\rho c\frac{\partial T}{\partial t}=k(\frac{\partial^{2}T}{\partial x^{2}}+\frac{\partial^{2}T}{\partial y^{2}}+\frac{\partial^{2}T}{\partial z^{2}})+Q$$

where ρ is the material density, c is the specific heat thermal conductivity, k is thermal conductivity, Q is mainly the latent heat of phase transition.

### 3.3. Metallurgical and Mechanical Analysis

**Figure 7:**
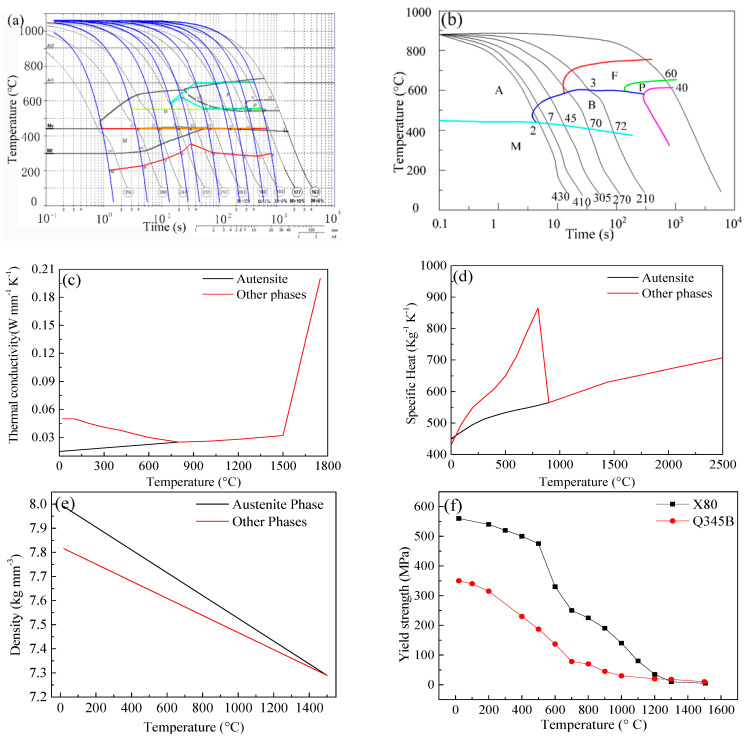

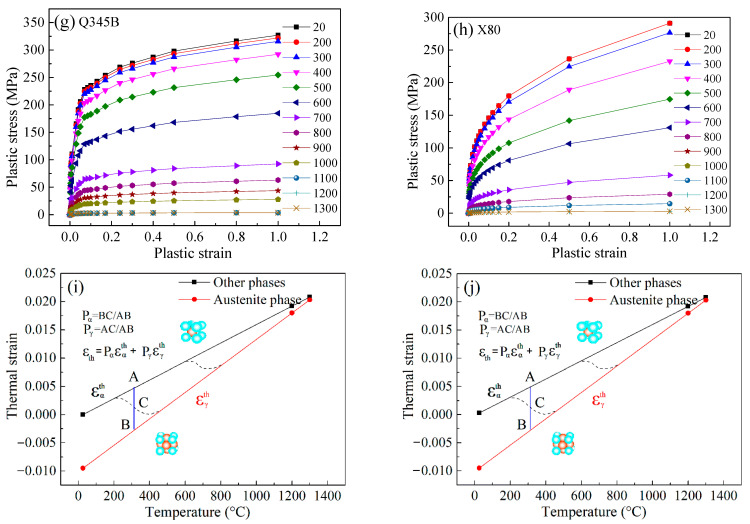
The material properties: (**a**) CCT diagram of X80; (**b**) CCT diagram of Q345B; (**c**) thermal conductivity, (**d**) specific heat, and (**e**) density of X80 and Q345B; (**f**) yield strength; (**g**) plastic strain-stress curve of Q345B; (**h**) plastic strain-stress curve of Q345B X80; (**i**) thermal strain of Q345; (**j**) thermal strain of X80.

Generally, the diffusion-controlled and the shear-type transformations were considered in a developed TMM model based on SYSWELD. The Johnson–Mehl–Avrami–Kolmogorov equation was used to describe the diffusion-controlled transformations, including the austenite-ferrite and the austenite-bainite transformations [19,28]. This phase transformation type was reproduced using the continuous cooling transformation (CCT) or isothermal transformation (IT) diagrams in SYSWELD. The CCT diagram of X80 was shown in Figure 7a.

The other material properties of X80 steel were also shown in Figure 7. The Koistinen–Marburger equation was used to describe the austenite-martensite transformation [29], and the governing equation was expressed as follows:

(6) $$P(T)=1-\exp[-b\times (M_{s}-T)]$$

where P is the martensite percentage, $M_{s}$ is the start temperature of martensite, b(0.029) is the law parameter.

For mechanical analysis, the governing equation of the total strain was given below:

(7) $$\varepsilon_{total}=\varepsilon_{e}+\varepsilon_{p}+\varepsilon_{th}+\varepsilon_{tp}$$

where $\varepsilon_{e}$ is the elastic strain, $\varepsilon_{p}$ is the plastic strain, $\varepsilon_{th}$ is the thermal and metallurgical strain rate, $\varepsilon_{tp}$ is the transformation-induced plastic strain.

In addition, the isotropic hardening model was employed in this investigation. The von Mises criterion described the yield behavior during welding. Poisson’s ratio remains a constant of 0.33 in the numerical simulation.

## 4. Results and Discussions

### 4.1. Temperature and Microstructure Fields Analysis

**Figure 8:**
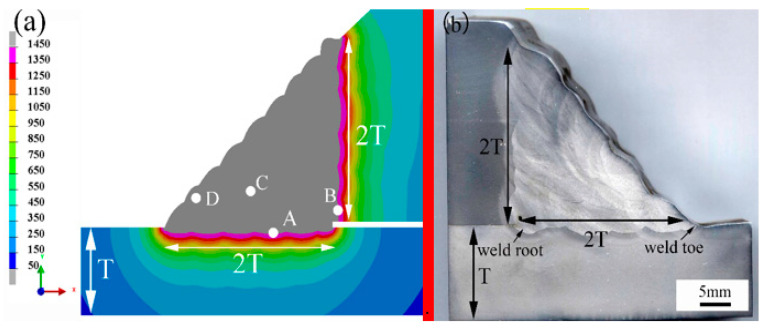
Weld morphology of the predicted vs. measured results (points of A–D are used to mark the position of thermal cycle points): (**a**) simulation results, (**b**) experimental results.

The reported works of literature [30,31] about sleeve repair welding mainly focused on the low-strength pipeline steel. Relatively few investigations on X80 pipeline repair based on experimental and numerical methods were reported. This paper obtained the temperature, microstructure, and stress distribution, by combining TMM (thermal-metallurgical-mechanical) simulations and corresponding verification experiments. In the case where the fillet weld consists of 25 welding passes and the fillet weld size was 2.0 times the X80 pipe’s wall thickness, the transverse cross-sectional weld joint was obtained and etched using 4% Nital solution, in order to verify the temperature simulation accuracy. During multi-pass welding, it was very difficult to obtain the welding pass morphology individually for each pass. In Figure 8, the peak temperature of each point was displayed in one picture. In this way, the numerical and experimental weld profiles can be compared directly. The welding porosity was found in Figure 8, and it was mainly affected by the stability of the molten pool. Moreover, the groove shape and welder technique have important effects on the stability of the molten pool. At present, shielded metal arc welding (SMAW) was still the primary welding technique during the sleeve repair welding process. Welding porosity was often found in the weld joint and to be difficult to avoid completely during SMAW. However, welding porosity did not mean wrong welding conditions or parameters. For quantitative evaluation, the penetrations of the overlaying welding beads were measured. The statistical results are listed in Table 4.

**Figure 9:**
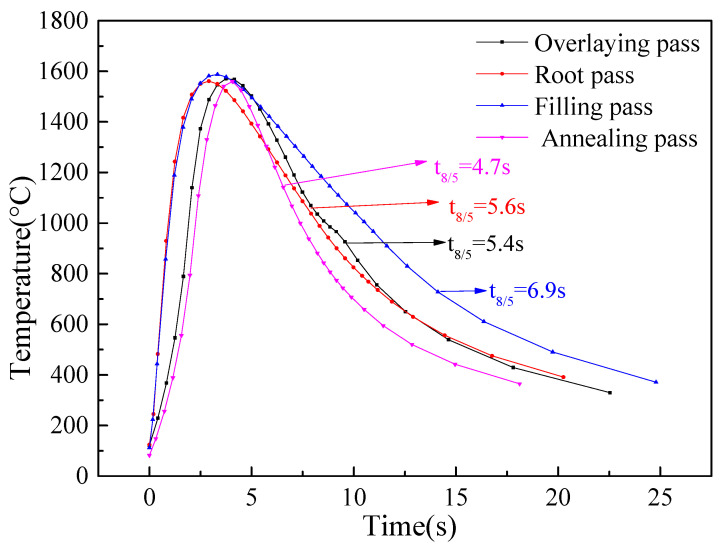
Welding thermal cycles.

The results found that the welding thermal cycles were mainly affected by the welding process and the cooling conditions. In this study, four specific points (A, B, C, D), as marked in Figure 8, were selected to exhibit the typical thermal cycles of the in-service sleeve repair welding. Points A and B were located at the heat-affected zones of the overlaying pass and the root pass, respectively. Points C and D were located at the weld zones of the filling pass and the temper pass, respectively. The thermal cycles were shown in Figure 9. The figure showed that the overlaying and the temper passes offered the fastest cooling rates. In consideration of the welding burn-through risk, these passes were usually welded with small heat inputs. Meanwhile, the flowing natural gas showed the most significant effect on these welding passes. It was found that, despite the root welding suffering worse cooling conditions, a larger heat input could significantly reduce its cooling rate. In contrast, the filling pass showed a relatively larger t_8/5_ time and a slower cooling rate.

**Figure 10:**
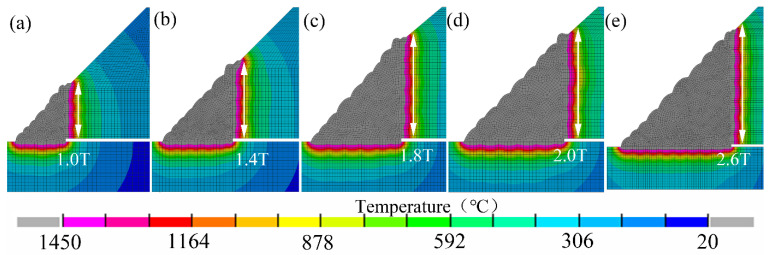
Welding temperature distributions: (**a**) 1.0 T, (**b**) 1.4 T, (**c**) 1.8 T, (**d**) 2.0 T, (**e**) 2.6 T.

Figure 10 showed the temperature distributions of the weld joints. The weld numbers mainly dominated the range of temperature fields.

**Figure 11:**
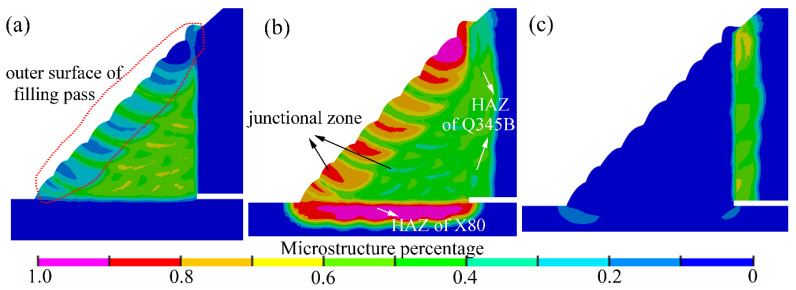
Numerical microstructure distribution contour: (**a**) ferrite, (**b**) bainite, (**c**) martensite.

**Figure 12:**
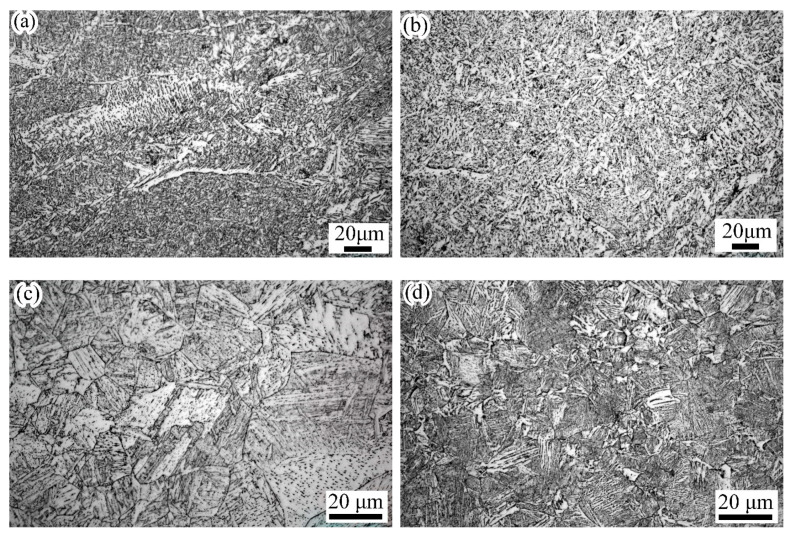
Microstructure: (**a**) junctional zone among welding pass, (**b**) weld zone, (**c**) HAZ located at X80 pipe, (**d**) HAZ located at Q345B pipe.

Furthermore, the microstructure distributions revealed the effects of the welding process, cooling conditions, and the material. Based on the numerical simulations, the microstructure distributions in the weld joint could be predicted and directly observed. As illustrated in Figure 11, the fillet weld zone mainly consisted of ferrite and bainite. The results showed that the temper and annealing passes showed a faster cooling, rate due to the welding process and the heat input discrepancies. Hence, the bainite percentage was relatively larger than the other passes. As shown in Figure 11, the microstructure located at the junctional zone and the central area of the welding passes exhibited a distinct difference. Moreover, the microstructure in the junctional zone showed a pronounced heterogeneity. The same phenomenon is also observed in the experimental microstructure results, as shown in Figure 12a. In the heat-affected zone (HAZ) of the X80 pipe, the microstructure was dominated by bainite. Moreover, a small amount percentage of ferrite was found in this area. The HAZ, located at the sleeve pipe, mainly consisted of bainite and martensite.

**Figure 13:**
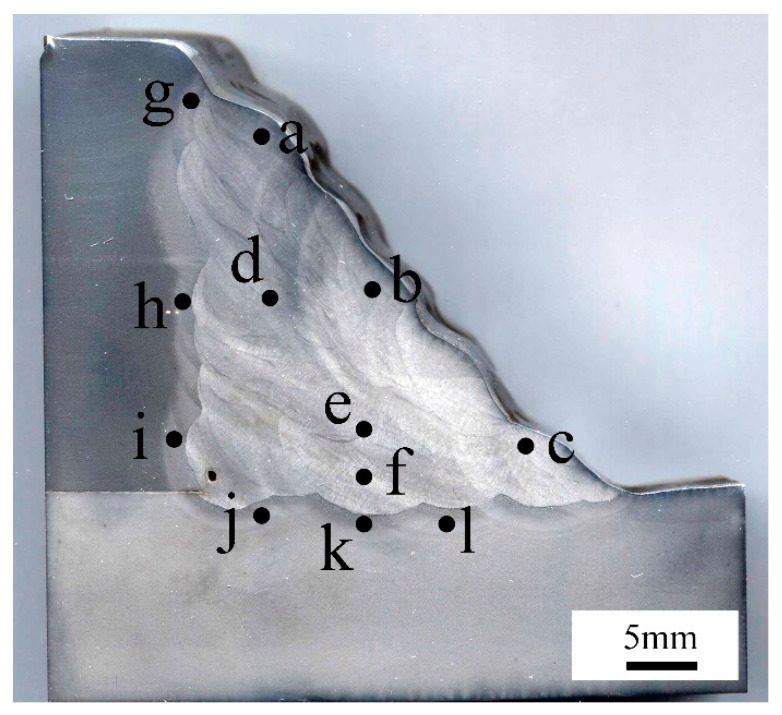
Hardness test point distribution (a–l marked the hardness test points).

The hardness of the welding joint was measured using a Vickers hardness tester (HVS-50, Beijing ShiDaiZhiFeng Instrument Co., Ltd., Beijing, China), with a force of 10 kgf (HV10) and a dwell time of 10 s. The hardness tests were used to verify the accuracy of the microstructure prediction. The test points (a–l) of the hardness tests are marked in the Figure 13.

According to the results of hardness tests as shown in Table 5, it was found that the hardness of the filling pass ranges from 239.8 (HV_10_) to 255.1 (HV_10_). Due to the microstructure discrepancies, the filling pass’ hardness located at the fillet weld’s outer surface ranges from 255.3 (HV_10_) to 270.3 (HV_10_). Furthermore, the experimental hardness results matched the microstructure distribution trend that was predicted by SYSWELD well. The hardness of X80 HAZ was about 279.5–289.7 (HV_10_). According to the experimental CCT diagrams and the hardness results, it was observed that the predicted microstructure distribution was reasonable. Furthermore, when the fillet weld size changed, the microstructure distribution was found to be similar to the results of Figure 11.

In this study, the microstructure was also observed by an optical microscope. As shown in Figure 12, the microstructures of the weld zone mainly consisted of side-plate ferrite, pro-eutectoid ferrite, acicular ferrite, and granular bainite. As demonstrated by numerical results, the phenomenon of microstructure heterogeneity was found in the junctional zone of welding passes, as shown in Figure 12a. In Figure 12c, the HAZ of X80 pipe mainly consisted of granular bainite and lath bainite. In Figure 12d, the HAZ of the Q345B pipe mainly consisted of lath bainite and lath martensite, and granular bainite.

### 4.2. Effects of Fillet Weld Size on Welding Residual Stress

It was widely accepted that tensile stress was harmful to the safety and integrity of welded structures. From the fracture mechanics perspective, the residual tensile stress could drive the nucleation and initiation of cracks. Therefore, as one of the favorable factors of hydrogen-induced cracking, more attention should be paid to the welding residual stress effects. Five simulation cases were investigated about the effects of the fillet weld size on the welding residual stress, as listed in Table 6. In these simulation cases, pipeline pressure was set as 7 MPa. The residual stress results were compared in an in-service state with a pipeline pressure of 7 MPa.

The results found that the restraint intensity in the hoop direction was larger than the axial direction. In general, the peak value of hoop residual stress usually reached the material’s yield strength. In practice, the hoop (longitudinal) residual stress was often larger than the yield strength. The reasons for this could be summarized as follows. Firstly, the welding microstructures in the HAZ and the weld zone were no longer the same as the base material after the welding. Furthermore, the yield strengths of these areas were significantly improved. Secondly, the mechanical behavior during the simulation was described by the true stress-strain curve rather than the nominal stress-strain curve. Thirdly, the material suffered from rapid heating and cooling processes. During this process, strain hardening occurred, and improved the yield strength. Lastly, the isotropic hardening model was widely used during the numerical simulations. Moreover, the isotropic hardening model would over-estimate the residual stress [32].

**Figure 14:**
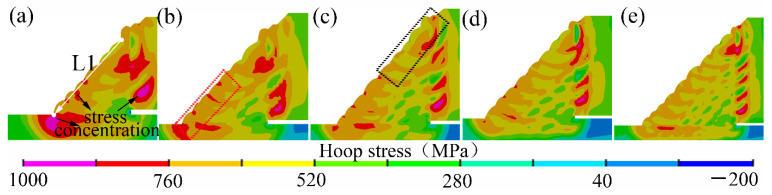
Hoop residual stress distribution: (**a**) case A, (**b**) case B, (**c**) case C, (**d**) case D, (**e**) case E.

**Figure 15:**
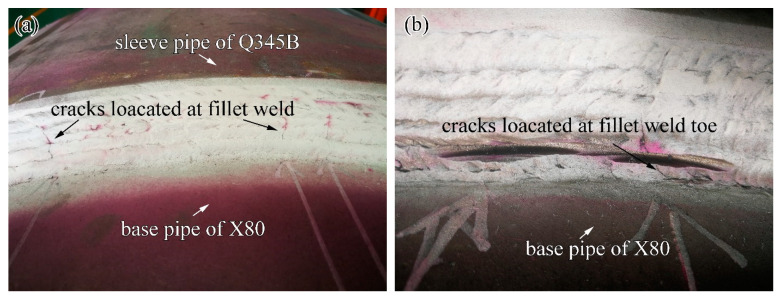
Fillet weld cracks in the weld zone: (**a**) cracks found on the outer surface, (**b**) cracks at the weld toe.

As shown in Figure 14, it was found that, as the fillet weld size increased, the peak value of the hoop residual stress decreased, and the stress concentration in the weld toe also decreased significantly. Furthermore, it was found that the stress concentration around the weld root reduced with the increase of the fillet weld size. After the in-service sleeve repair welding, the fillet weld cracks in the weld zone were usually in the axial direction, which was perpendicular to the hoop residual stress, as given in Figure 15. The crack perpendicular to the hoop stress was one of the significant problems in the girth weld joint. These cracks usually originate from the weld root, weld toe, and the outer surface of the fillet weld between welding passes. Zerbst [33] and Schork et al. [34] demonstrated that weld surface roughness significantly affected crack initiation. Weld re-solidification ripples on the weld surface and weld ripples between welding passes or near the weld toe could cause stress concentration and the corresponding crack initiation. These findings provided good support for our research results. The external surface cracks in the weld zone were usually found in the red rectangular-shaped region near the X80 pipe, marked in the dashed line, as shown in Figure 14b. From Figure 14b,c, it was observed that the hoop stress in the red rectangular-shaped region was larger than that in the black rectangular-shaped area. Furthermore, the stress concentration located at the outer surface of the fillet weld between the welding passes was also found based on the numerical results. The hoop stress showed a correlation with the welding cracks. From the perspective of the welding residual stress, the fillet weld size reduction was found to be harmful to the safety and integrity of the in-service welded structures. From the viewpoint of welding residual stress, fillet weld size was suggested to be larger than 1.4 T.

**Figure 16:**
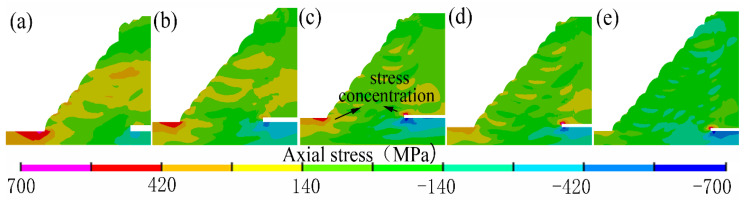
Axial residual stress distribution: (**a**) case A, (**b**) case B, (**c**) case C, (**d**) case D, (**e**) case E.

Figure 16 showed the distribution contours of the axial residual stresses on the middle transverse cross-section of the fillet weld joint. It was found that the stress concentrations that were induced by the geometrical shape changes were found in the locations of the weld toe and the weld root. Furthermore, it was found that, with the increase of the weld fillet size, the peak value of the axial residual stress decreased, and the stress distribution became uniform. It was also found that the stress concentration was reduced.

**Figure 17:**
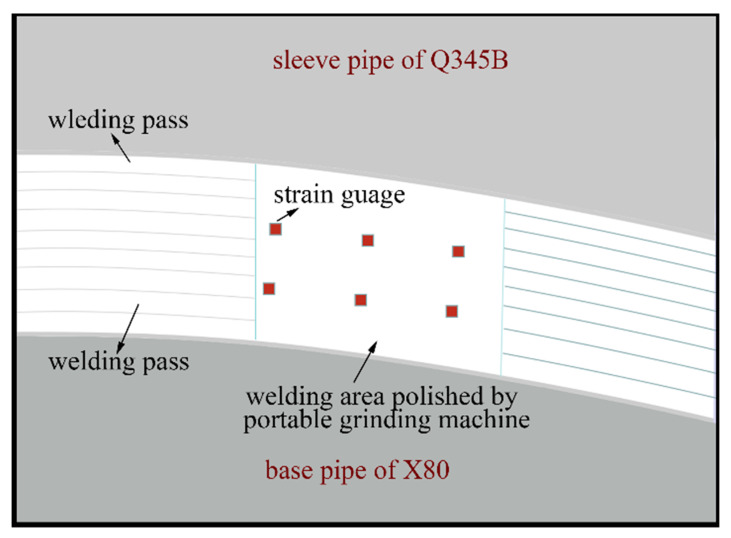
Schematic diagram of residual stress measurement.

Compared to the non-destructive residual stress methods, the hole-drilling strain gauge method (HDM) was usually reliable and economical. In this research, HDM was chosen to characterize the residual stress distribution at the fillet weld zone. A schematic diagram was provided, as shown in Figure 17. Before residual stress measurement, the surface of the fillet weld zone was carefully polished. Thereafter, the strain gauges were stuck to the polished area. In practice, residual stress in the same welding pass usually had the same or similar values. The simulation results and theory also supported this law. This research arranged three or more strain gauges at the same welding pass to obtain reliable results, as shown in Figure 17. When two or more measurement values were similar, these values would be regarded as reliable results, and the average value was adopted in our research.

**Figure 18:**
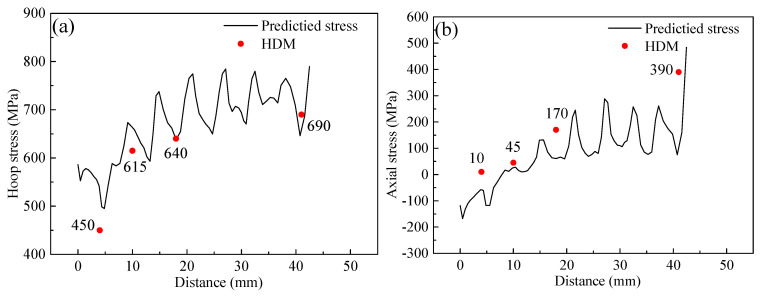
Residual stress predicted by SYSWELD vs. measured by HDM: (**a**) hoop stress, (**b**) axial stress.

In the case of D, the residual stress of along L1, as marked in Figure 14, was measured to verify numerical simulation’s prediction accuracy. As shown in Figure 18, the residual stresses in the hoop and axial directions correlated well with the experimental results.

### 4.3. Effects of Sleeve Pipe Material on Microstructure and Residual Stress Distributions

Based on the stress analysis in Section 4.2, it is found that the welding residual stresses in and near the X80 pipe are larger than those in and near the sleeve pipe. The discrepancies in materials and restraint levels might be a reason for this phenomenon. In order to clarify this phenomenon and investigate the effects of the sleeve pipe’s materials on the stress distribution, in this study, the material of the sleeve pipe was replaced by X80 steel. Based on SYSWELD, the microstructure and the stress results were compared.

**Figure 19:**
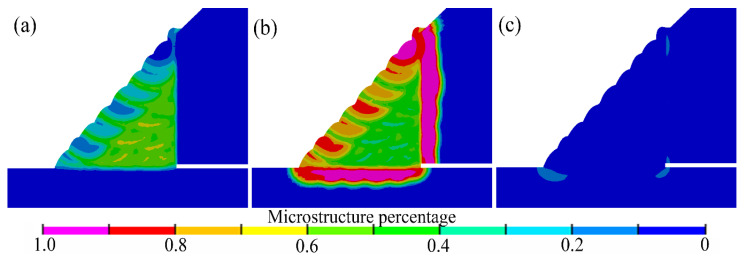
Microstructure predicted by SYSWELD: (**a**) ferrite, (**b**) bainite, (**c**) martensite.

Note that the X80 pipe was usually defined as the base pipe, and the Q345B pipe was usually defined as sleeve pipe, as shown in Figure 1. The microstructure contours were shown in Figure 19. When compared with Figure 11, the microstructure in fillet weld and the HAZ of X80 was found to remain similar to those in Figure 11. In Figure 19b, the HAZ of sleeve pipe was dominated by bainite after replacing the material with X80. As shown in Figure 19c, a very small amount of martensite was found in the HAZ of the sleeve pipe.

**Figure 20:**
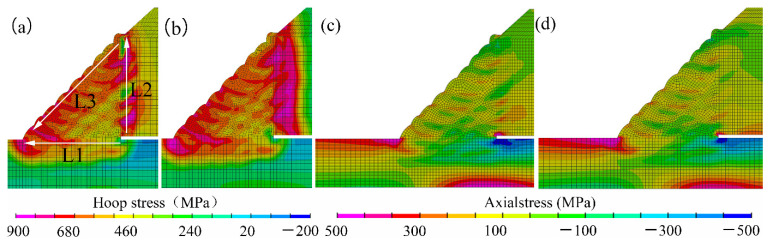
Effect of sleeve pipe material on residual stress distribution: (**a**) Q345B, (**b**) X80, (**c**) Q345B, (**d**) X80.

After replacing sleeve material with X80, the welding residual stress contours were given in Figure 20. As shown in Figure 20a,b, when X80 replaced the sleeve pipe material, hoop stresses in the weld zone, base pipe, and sleeve pipe became larger in general. As shown in Figure 20c,d, the stress concentration at the weld toe was also found to be increased. Furthermore, the sleeve pipe material replacement showed an insignificant effect on the axial stress distribution. During in-service welding, the welded structure was subjected to the multiaxial stress state, severe restraints in both hoop and axial directions. Zerbst [35] reported that the residual stress in this research could be treated as a mixture of medium- and long-range stress. The long-range stress was also called the “reaction stess”, which was affected by restraint and external load stress, and the medium-range residual stress was featured with the local plastic deformation. In this research, the welding residual stress of the sleeve-repaired welded joint also showed the typical characteristics of medium-range stress [35]. The geometry change of the fillet weld joint and local plastic deformation near the weld toe showed a pronounced effect on the residual stress distribution, as exhibited in Figure 20.

As illustrated in Figure 20, the stress concentrations in the weld toe and the weld root were found to become more significant after replacing Q345B with X80. Generally, the hoop stress in the sleeve pipe increased, and the values became larger than those in the base pipe. In the fillet weld zone, the residual stress near the base pipe was still larger than that near sleeve pipe. This phenomenon was mainly caused by the restraint level discrepancy and geometrical shape change [36]. The effects of material strength on the welding residual stress near the weld toe were also investigated by Zerbst [35] and Farajian [36]. The weld zone was manufactured in a tensile test sample, whereas the effects of material strength on welding residual stress distribution were discussed. With the increase of material strength, tensile residual stresses levels around the weld zone and weld toe were significantly improved, while the residual stress magnitude away from the weld zone still remained at a low level. Hence, it could be concluded that the increase of material change would improve the stress concentration caused by geometry change. After material replacement, the restraint level was also improved. In addition, the crack-resistant ability of X80 usually was worse than Q345B. From the viewpoint of welding stress, the cracking risk in the base pipe, weld zone, and sleeve pipe will became larger than the traditional repair welding process.

**Figure 21:**
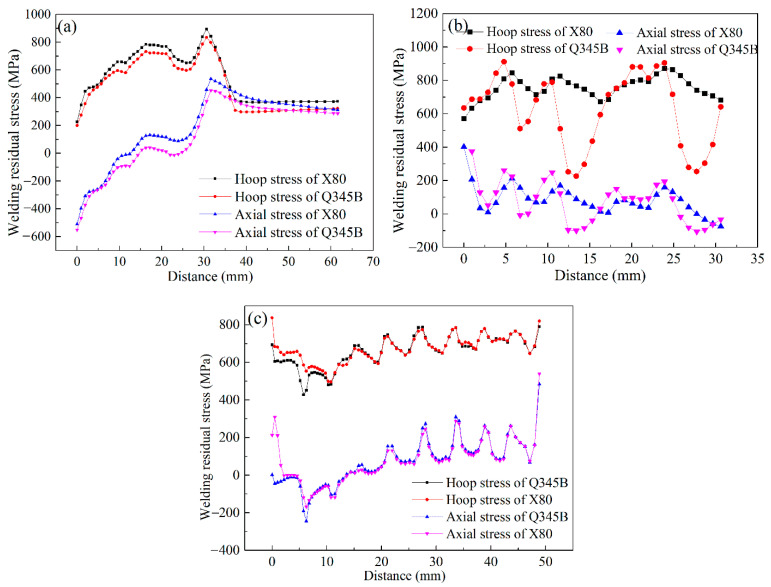
Residual stress distribution along (**a**) L1, (**b**) L2, (**c**) L3.

The stress distribution curves along L1, L2, and L3 were drawn in Figure 21. Note that L1, L2, and L3 have been marked in Figure 20a. After replacing the sleeve pipe material with X80, the residual stresses in the base and sleeve pipe were found to become larger, as shown in Figure 21. As illustrated in Figure 21c, the stress concentrations at the weld toes were found to be increased.

## 5. Conclusions

In this work, a TMM numerical simulation based on SYSWELD was developed. The experimental tests were conducted to obtain the temperature-microstructure-stress field distributions after in-service sleeve repair welding. The effects of the fillet weld size and the sleeve material strength on the residual stress distribution and the structural safety were discussed. The main conclusions were drawn as follows:

(1) The heat dissipation between the outer surface and air was usually treated as the natural convective heat transfer, and the heat dissipation between the X80 pipe’s inner surface and the flowing natural gas was defined as the forced convection heat dissipation. The temperature distribution and the weld pass size matched well with the experimental results. The t_8/5_ of the in-service repair welding ranged from 4 s to 7 s.

(2) When the material of the sleeve pipe was Q345B, the fillet weld zone was composed of ferrite and bainite. Furthermore, the HAZ of sleeve pipe was composed of lath martensite, lath bainite, and granular bainite. Moreover, the HAZ of the X80 pipe was mainly composed of granular bainite and a small amount of ferrite. The microstructure was predicted based on the TMM model and verified using metallographic observation and hardness tests. It was observed that the numerical results correlated well with the experimental results.

(3) It was found that, as the fillet weld size increased, the peak value of the hoop residual stress decreased, and the stress concentration in the weld toe significantly decreased. The hoop stress at weld toe reduced from ~860 MPa of case A to ~680 MPa of case E. Furthermore, the axial stress concentration was found to be reduced as the fillet weld size increased. The axial stress at weld toe reduced from ~440 MPa of case A to ~380 MPa of case E. From the viewpoint of welding residual stress, fillet weld size is suggested to be larger than 1.4 T.

(4) When the sleeve pipe material was Q345B, and base pipe material was X80, the cracks were mainly found in the weld toe, and the weld area near the X80 pipe. Weld re-solidification ripples on the weld surface and weld ripples between welding passes or near the weld toe can cause stress concentration and the corresponding crack initiation. The stress concentration showed a correlation with the cracking behavior. The stress concentration and the high-level residual stress were mainly affected by the interaction of the weld joint’s geometrical morphology, restraint level, and material.

(5) When the material of the sleeve pipe was changed from Q345B to X80, the high-level tensile stress zone was found to be enlarged, and the stress concentration in the weld toe increased. The hoop stress at weld toe increased from ~750 to ~800 MPa, and the axial stress at weld toe increased from ~500 to ~600 MPa. Traditionally, the cracks are mainly found in the weld zone and weld toe. After implementing the new sleeve repair welding process where X80 replaces the material of the sleeve pipe, the cracking risk in sleeve pipe will improve. From the viewpoint of welding stress, the cracking risk in the base pipe, weld zone, and sleeve pipe will become larger than the traditional repair welding process.

## Figures and Tables

**Table 1:** Chemical compositions (in wt%) of X80, Q345B and E5515-G deposited metal.

Material	C	Si	Mn	S	P	Cr	Ni	Mo	V
X80	0.12	0.45	1.85	0.025	0.015	--	0.013	--	<0.012
Q345B	0.20	0.55	1.48	0.013	0.0071	--	--	--	--
E5515-G	0.090	0.45	1.81	0.013	0.0081	0.034	0.018	0.18	0.010

**Table 2:** The mechanical properties of X80, Q345B and deposited metal of E5515-G.

Material	Tensile Strengh (MPa)	Yield Strength (MPa)	Elongation (%)	Charpy Impact Energy (J)
X80	625–825	555–690	≥14.5	--
Q345B	≥510	≥345	≥21	>34 (0 °C)
E5515-G	≥550	≥460	≥17	102, 96, 94 (−30 °C)

**Table 3:** Welding parameters.

Welding Passes	Welding Voltage (V)	Welding Current (A)	Welding Speed (cm/min)	Heat Input (KJ/mm)
Overlaying/temper pass	22–28	100–130	10–16	0.7–1.7
Root pass	22–28	100–130	6–15	0.7–3.0
Filling pass	22–28	100–130	6–15	0.7–3.0
Annealing pass	22–28	100–130	10–15	0.7–1.7

**Table 4:** Numerical and experimental penetrations of the overlaying welding beads.

Welding Pass Number	1	2	3	4	5
Numerical penetration (mm)	1.2	1.25	1.3	1.28	1.15
Experimental penetration (mm)	1.3	0.5	1.6	1.32	1.07

**Table 5:** Hardness value.

Point	a	b	c	d	e	f
Hardness (HV_10_)	277.3	254.9	255.3	250.8	239.8	255.1
Point	g	h	i	j	k	l
Hardness (HV_10_)	289.7	285.7	279.5	279.5	269.6	272.3

**Table 6:** Simulation cases.

Case	A	B	C	D	E
Welding fillet size	1.0 T	1.4 T	1.8 T	2.0 T	2.6 T

Note: T (15.3 mm) is the wall thickness of the X80 pipe.

## Data Availability

Not applicable.
